# Low/High Multi‐Frequency Stimulation of the Subthalamic Nucleus Improves Verbal Fluency Maintaining Motor Control in Parkinson's Disease

**DOI:** 10.1002/mds.30254

**Published:** 2025-06-11

**Authors:** Lucia Ricciardi, Francescopaolo Cucinotta, Elena Pegolo, Arturo Abundes‐Corona, Bryony Ishihara, Israt Hossain, Zimi Sawacha, Michael Hart, Erlick Pereira, Francesca Morgante, Alfonso Fasano

**Affiliations:** ^1^ Neurosciences and Cell Biology Institute Neuromodulation and Motor Control Section, City St George's University of London London United Kingdom; ^2^ Department of Neurology University of California San Francisco San Francisco California USA; ^3^ Edmond J Safra Program in Parkinson's Disease, Morton and Gloria Shulman Movement Disorders Clinic, Toronto Western Hospital, UHN Toronto Ontario Canada; ^4^ Division of Neurology University of Toronto Toronto Ontario Canada; ^5^ Department of Information Engineering University of Padova Padova Italy; ^6^ Krembil Brain Institute Toronto Ontario Canada

**Keywords:** cognition, deep brain stimulation, non‐motor symptoms, Parkinson's disease, verbal fluency

## Abstract

**Background:**

High frequency deep brain stimulation of the subthalamic nucleus (STN‐DBS) is a well‐established therapy for Parkinson's disease (PD) motor symptoms, however, its effect on non‐motor symptoms is controversial. Low frequency DBS can improve cognition, but its effects on motor functions are detrimental.

**Objective:**

Our goal was to evaluate the effect on verbal fluency (VF) of dual frequency STN‐DBS combining high and low frequency (130 + 10 Hz) as compared to 130 Hz or 10 Hz alone and to OFF stimulation. The effect on motor symptoms, working memory, and subjective feelings was also assessed.

**Methods:**

We used a randomized order of experimental conditions with a double‐blind design to assess the effects of 130 Hz, 10 Hz, and 130 + 10 Hz stimulation as compared to OFF stimulation in 18 PD patients with STN‐DBS. In each condition, participants completed: phonemic and action VF, N‐back task, and visual analogue scales for fatigue and stress level. Motor functions and gait velocity were also assessed.

Friedman analysis of variance were conducted to determine whether change scores from baseline OFF stimulation, in our primary (VF) and secondary outcomes measures (motor functions, N‐back task, subjective feelings) were different in the three stimulation conditions.

**Results:**

VF improved more in the 130 + 10 Hz condition than 130 Hz condition (*P* = 0.006); there was no difference between 130 + 10 Hz and 10 Hz (*P* = 0.2) and between 130HZ and 10 Hz (*P* = 0.6). There was a significant difference among the stimulation conditions for the motor score (χ^2^(2) = 11.1, *P* = 0.004), it being worse at 10 Hz than 130 Hz (*P* = 0.002) and 130 + 10 Hz (*P* = 0.01).

**Conclusions:**

Dual frequency STN‐DBS improves phonemic VF while maintaining a beneficial effect on motor signs of PD. © 2025 The Author(s). *Movement Disorders* published by Wiley Periodicals LLC on behalf of International Parkinson and Movement Disorder Society.

Deep brain stimulation (DBS) of the subthalamic nucleus (STN) or globus pallidus pars interna is an established surgical intervention for Parkinson's disease (PD), successfully improving motor symptoms.^1^, ^2^ Yet the effects of DBS on non‐motor symptoms remain debated. Although there is increasing evidence that DBS can improve symptoms like sleep and pain, its impact on neuropsychological symptoms continues to be variable.^3^, ^4^

Current clinical DBS parameters use high frequency stimulation (130–180 Hz) to treat PD motor symptoms. However, concerns have been raised about potential adverse effects of these conventional parameters on cognitive functions.^3^, ^5^ Meta‐analyses indicate that DBS, particularly STN‐DBS, is associated with a moderate worsening of both phonemic and semantic verbal fluency (VF) in PD patients.^3^, ^6^ The link between cognition and VF is complex and still debated,^7^ with studies suggesting that VF is influenced by various cognitive processes, including executive function, working memory, processing speed, and language. The mechanisms behind the VF decline post‐DBS are still unclear, and evidence from randomized controlled trials on best medical treatment suggests that disease progression alone does not explain the VF deterioration in DBS patients.^5^, ^8^ Additionally, reductions in dopaminergic medication after surgery in STN‐DBS patients are unlikely to be responsible for this decline.^6^ Instead, factors related to the surgery or stimulation, likely influenced by electrode positioning, may be involved.^9^, ^10^, ^11^ However, the evidence remains inconclusive and limited to date.^3^, ^6^

Recent research has explored novel stimulation protocols, such as low‐frequency DBS (4–10 Hz), aimed at modulating neural circuits implicated in non‐motor symptoms and specifically in cognitive functions in PD.^9^, ^12^, ^13^, ^14^, ^15^, ^16^ For example, several studies found that 10 Hz STN‐DBS increases VF compared to both high‐frequency and no stimulation.^9^, ^12^ Notably, this improvement by 10 Hz stimulation is specific to episodic VF, with no change found for non‐episodic VF. Some studies have also shown an improvement with 4 to 5 Hz DBS in executive functions tested with the Stroop Test^14^, ^17^ and in working memory assessed with a Stenberg task.^15^ The effect on working memory was particularly evident in patients with lower baseline performance and was linked to increased connectivity between the STN and the right middle frontal gyrus, suggesting that specific neural pathways mediate these cognitive benefits.^15^ Moreover, recent research has shown an effect of acute 10 Hz DBS on emotional processing in PD.^18^, ^19^

Although low‐frequency stimulation has shown promise for improving cognitive and emotional outcomes, it is important to note that frequencies below 50 Hz may actually exacerbate or even induce parkinsonism.^20^, ^21^, ^22^ For example, 10 Hz stimulation has been associated with pathologic oscillatory networks related to PD tremor.^23^ For this reason, the clinical application of low frequency DBS is limited.

In this study, we will focus on VF with the primary objective of investigating the effect of concomitant 130 Hz and 10 Hz stimulation of the STN using two different contacts of the same electrode. The secondary objectives are to evaluate the effects of concomitant stimulation at 130 Hz and 10 Hz on motor function. The contributing effects of attention/working memory as well as the subjective feelings of fatigue and stress on the primary outcome measure (VF) have been also accounted for.

We hypothesize that this dual stimulation may optimize both VF and motor outcomes, potentially leading to a more comprehensive therapeutic strategy for PD.

## Materials and Methods

### Study Design

We used a randomized order of experimental conditions with a double‐blind design to assess the effects of a novel dual frequency stimulation (stimulating simultaneously in the same electrode a superior contact with 130 Hz and an inferior contact with 10 Hz) as compared to the conventional high frequency (130 Hz) and low frequency (10 Hz) DBS alone. In the dual frequency stimulation, the superior contact was the one used for clinical chronic treatment of motor symptoms; the inferior contact was the one below it. Both the patients and the raters assessing their cognitive and motor performances were masked to the experimental conditions.

Subjects were recruited at the movement disorders center of St George's Hospital in London, United Kingdom (n = 13), and of Toronto Western University Hospital, Toronto, Canada (n = 5).

### Study Participants

Inclusion criteria included a clinical diagnosis of idiopathic PD, without a history of other neurological or psychiatric disorders; patients implanted for a minimum duration of 6 months with a bilateral STN‐DBS; a DBS device enabling different frequencies stimulations in different contacts (Vercise, Cartesia X, Boston Scientific Corp, Valencia, CA); clinically defined best stimulation settings involved one of the superior contacts bilaterally (second level of stimulation and above); participants could stand unaided and walk without an assistive device in their usual *on* medication/ON stimulation condition and could give written consent.

At the study time, DBS settings and medications had been optimized and stable for at least 3 months.

### Electrode Placement

Leads and contacts position was confirmed using postoperative imaging in native space using the Lead‐DBS toolbox version 3.0 (https://www.lead-dbs.org/) and imported into MATLAB R2023a (The MathWorks, Natick, MA). Specifically, computed tomography scans containing lead location information were co‐registered to pre‐operative T1‐weighted magnetic resonance imaging (MRI) using advanced normalization tools, then transformed into MNI space. For electrode reconstruction, leads were first automatically pre‐reconstructed using the PaCER toolbox,^24^ then were manually refined on visual inspection.

Following electrode localization, volume of tissue activations (VTAs) corresponding to each stimulation condition were generated for each patient using finite element modeling. The 130 Hz condition, which targeted more superior contacts, and the 10 Hz condition, which targeted more inferior contacts, were modeled separately. For the dual frequency condition, both the dorsal and ventral stimulation parameters were incorporated as distinct sources within the model. Because frequency parameters cannot be directly incorporated into the VTA, this analysis was designed to assess whether differences in the spatial location of stimulation between conditions influenced VF outcomes.

### Screening, Baseline Testing, and Randomization

Pre‐screening was done in the movement disorders clinics and via research databases. Participants who met the criteria underwent baseline assessments including: gathering of demographic and clinical information; an evaluation of overall cognitive functions using the Montreal Cognitive Assessment (MoCA)^25^; an evaluation of gait and falls with the new freezing of gait questionnaire.^26^ Levodopa‐equivalent daily dose (LEDD, mg/day) was computed according to.^27^

### Experimental Protocol

The experimental protocol is summarized in Supplementary Figure S1.

Patients were assessed under their regular dose of PD medications (*on* medication) during a single‐visit protocol during which we applied bilateral STN‐DBS. At baseline patients underwent the assessment OFF stimulation. Three stimulation conditions were then chosen in a randomized order: (1) conventional 130 Hz at the contact and with the parameters used for chronic stimulation, including the directional settings when used in standard care; (2) 10 Hz delivered at the contact inferior to the one used during condition 1; and (3) dual frequency stimulation combining condition 1 and 2. The amplitude and the pulse width were those used for chronic treatment and were maintained constant for conditions 1 and 2. During condition 3, two interleaved “areas” were activated per each hemisphere, each with the same amplitude and pulse width used in conditions 1 and 2 (but with different frequencies, high and low).

There was a 30‐minute washout period between conditions to minimize carryover effects from the previous condition. Patients were prompted to take their medications on time and the clinician ensured they were on for the whole testing time.

### Primary Outcome Measure: Verbal Fluency

Our primary outcome measure was VF (ie, the number of words in the phonemic VF task and the number of words in the action VF task). In detail, phonemic VF^28^ was assessed by asking the participants to name within 1 minute as many words as possible starting with a letter that was randomly assigned to control for letter‐specific biases (F, A, S, B, C, D, M, R). Consistent with previous studies, we selected letters with a homogeneous level of difficulty, excluding the more challenging ones (eg, J and U).^29^

Patients were told to avoid using proper names, numbers, places, and similar words (eg, walk/walking, run/running). The corresponding letter was provided verbally to each patient just before recording the task through a voice recorder. The number of words pronounced in 1 minute was counted by a masked assessor.

Action fluency^30^ was assessed by asking the participants: “I'd like you to tell me as many different things as you can think of that people do”. If the subject had difficulty understanding the task, the words “verb” or “action” were used for clarification. As above, the performance was recorded through a voice recorder and the total number of unique verbs generated in 1 minute was counted by a masked assessor.

### Secondary Outcome Measure: Motor Assessment

Secondary outcome measures were the Motor Composite Score (ie, the sum of Movement Disorder Society Unified Parkinson's Disease Rating Scale [MDS‐UPDRS] items 3.4, 3.10, 3.17 scores, assessing finger tapping, gait and rest tremor, respectively) and gait velocity, measured as number of meters walked/60 seconds.

Participants were video‐recorded at rest and while performing a finger tapping task for 20 seconds with the right and left hand. They then walked for 1 minute along a standardized 10 meters pathway at their own speed. A blinded assessor (L.R.) rated the tasks from the videos according to the MDS‐UPDRS 3.4 (finger tapping). 3.10 (gait). and 3.17 (resting tremor). A second blinded assessor (I.H.) calculated gait velocity from the video as follows: number of meters walked/60 seconds.

Moreover, participants were also required to perform a dual task where they walked along the same standardized pathway as above for 1 minute while performing a cognitive task (VF task using parallel forms from the single task to avoid learning effect) simultaneously. Gait velocity in the dual task was calculated by the same blind assessor and with the same method used for the gait velocity in the single task (number of meters walked/60 seconds).

### Assessment of Confounders

To control for cognitive and emotional confounders that might influence VF performance, attention/working memory, fatigue, and stress level were assessed.

Attention and working memory were assessed by means of a computer‐based N‐back task in which participants were asked to perform a 2‐back task implemented and presented online using the PsyToolkit platform.^31^, ^32^ Participants were presented a sequence of one‐by‐one stimuli and they had to decide if the current stimulus was the same as the one presented N (two) trials ago. They were required to press the letter “m” on the keyboard if the letter presented was the same as 2 letters before, otherwise withhold. They completed a practice block of 25 trials before the actual task began to assure full comprehension of the task. The task consisted of three blocks of 25 trials. Each stimulus was presented for 500 milliseconds and answers could be given in a 3‐second time window. Performances were evaluated considering the percentage of correct trials, matching trials, and false alarms over the three blocks.

Visual analogue scales ranging from 0 to 10 were used to assess fatigue and stress levels. In detail, subjects were asked to rate their level of (1) fatigue, from 0 = no fatigue at all/energetic to 10 = extremely fatigued; and (2) stress, from 0 = no stress at all/completely relaxed to 10 = extremely stressed.

### Statistical Analyses

To assess the effects of the three stimulation conditions on our primary and secondary outcome measures, changes in phonemic VF, action VF, Composite Motor Score, gait velocity, N‐back task scores, and fatigue and stress scores were calculated relative to baseline (OFF stimulation) performances. We chose to use data normalized relative to baseline to control for baseline differences among subjects. For each participant, the change score for each task and condition was determined by subtracting the score obtained during the stimulation condition from the baseline score (OFF stimulation) as follows:

Change score=score duringOFFstimulation−score during stimulation condition.

Differences in change scores for each outcome measure in the three different stimulation conditions (130 Hz, 10 Hz, and 130 + 10 Hz) were analyzed using Friedman test given the small sample size and the lack of normal distribution of the residuals. Post hoc tests were performed using Wilcoxon tests when the Friedman analysis of variance (ANOVA) indicated a significant main effect.

For the dual task we calculated for each subject the dual‐task cost for gait velocity (DTC) according to the formula^33^:

$$\mathrm{DTC}=\begin{array}{l} 100\times (\text{single}-\text{task gait velocity}-\text{dual}\\ -\;\text{task gait velocity})/\left(\text{single}-\text{task gait velocity}\right). \end{array}$$

Pearson or Spearman correlations were used according to the data distribution to explore the relationship between change score in phonemic VF and clinical data (age, disease duration, LEDD, MoCA, and baseline phonemic VF score) and change scores in fatigue, stress, and motor data.

A significance level of *P* < 0.05 was adopted for all the statistical analyses. All the analyses were performed in MATLAB (version R2024b) and SPSS (version 29).

## Ethical Approval

All patients provided written informed consent and ethical approval was obtained (London cohort, study protocol number 26894; Toronto cohort, study number CAPCR ID 24‐5314.0.1).

## Results

Eighteen PD patients with bilateral STN‐DBS were included. Supplementary Tables S1–S3 show demographic and clinical data of the study population. All patients were implanted with Boston Scientific devices: Vercise (directional lead with 1–3–3‐1 configuration) was used for all subjects except two who received Vercise Standard Lead (model 2201 with 8‐ring contacts) and one Cartesia X (leads with 3–3–3‐3‐3‐1 configuration).

Two patients could not tolerate the OFF‐stimulation condition: one experienced significant worsening of tremor and pain, the other reported severe pain. Therefore, they were excluded from further analysis, leaving a final sample of 16 participants.

Electrode placement inside the STN was confirmed in all participants (Supplementary Fig. S2).

### Phonemic Fluency

**FIG. 1:**
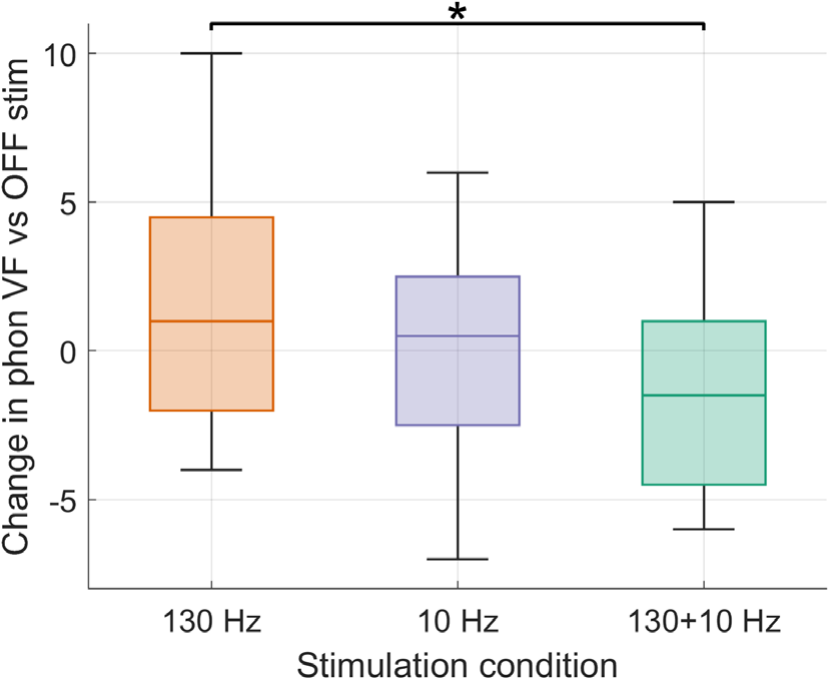
Effect of the different deep brain stimulation (DBS) conditions on phonemic verbal fluency (VF). Box plots show phonemic verbal fluency (VF) changes in the three stimulation conditions (130Hz, 10Hz, 130+10Hz) as compared to baseline VF OFF stimulation. VF is expressed in terms of difference of number of words with negative values representing an improvement with respect to the OFF condition. *p< 0.05. Abbreviations: VF; verbal fluency. [Color figure can be viewed at wileyonlinelibrary.com]

Analysis with Friedman ANOVA revealed a significant effect of stimulation condition on the change scores of phonemic VF (χ^2^(2) = 6.50, *P* = 0.026). Post‐hoc Wilcoxon tests showed that the participants' phonemic fluency improved more in the 130 + 10 Hz condition compared to the 130 Hz condition (*P* = 0.006) (Fig. 1). There was no significant difference between 130 + 10 Hz and 10 Hz (*P* = 0.2) and between 130 Hz and 10 Hz (*P* = 0.6).

There was no effect of the different stimulation conditions on change scores of action fluency (Supplementary Table S4).

### Motor Outcomes

**FIG. 2:**
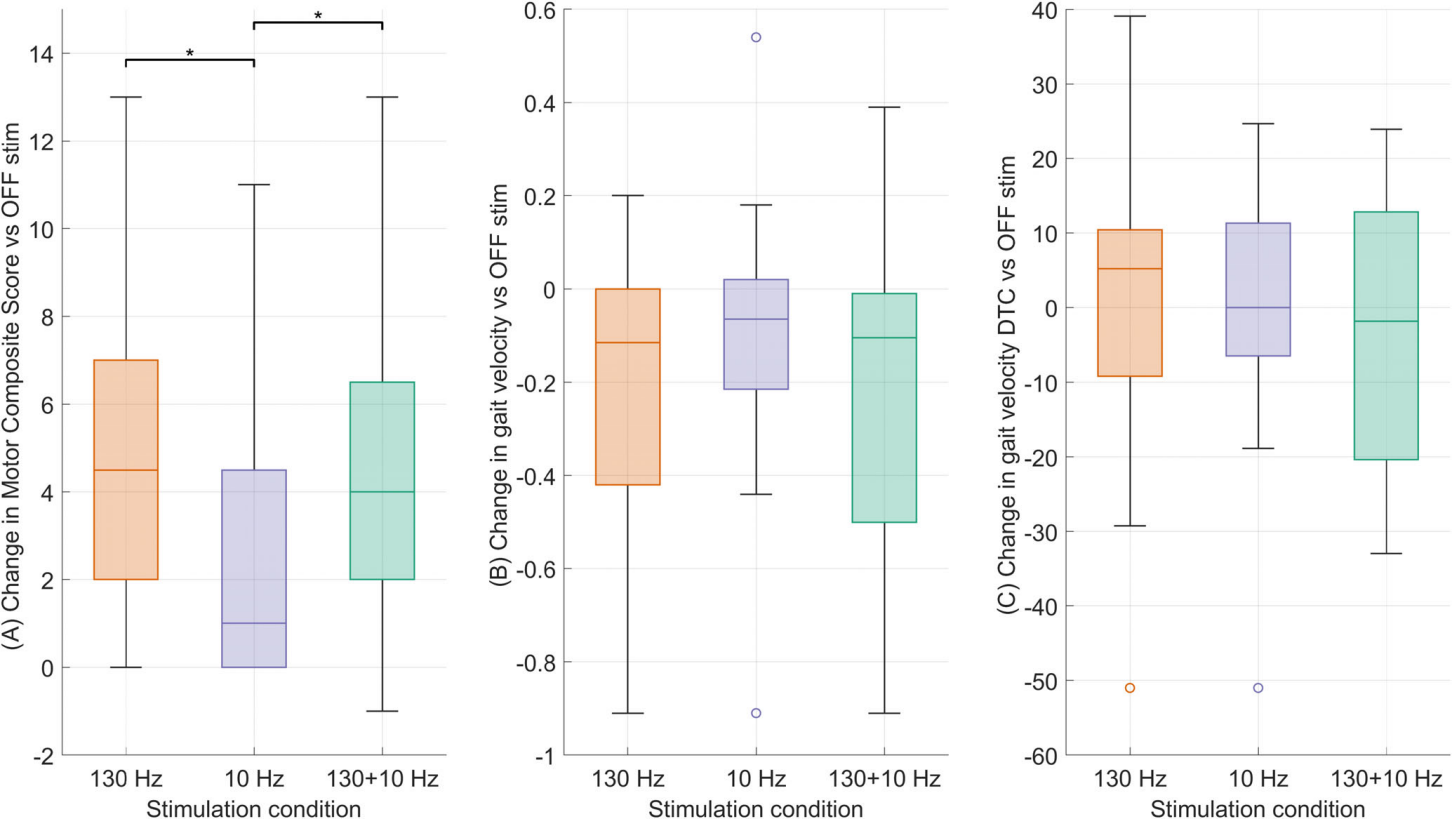
(**A**) Effect of the different deep brain stimulation (DBS) conditions on Composite motor score. Changes in the composite motor score in the three stimulation conditions (130 Hz, 10 Hz, 130 + 10 Hz) compared to the OFF condition are represented in the box plots. Composite motor score is computed as the sum of the values of MDS‐UPDRS 3.17 (resting tremor), 3.4 (finger tapping), and 3.10 (gait). Positive values identify improvements with respect to the OFF condition. *P < 0.05. (**B‐C**) Effect of the different DBS conditions on gait velocity. Box plots show changes in gait velocity in the single condition (B) and for the DTC (C) in the three stimulation conditions (130 Hz, 10 Hz, 130+10 Hz) compared to OFF stimulation. Negative values of gait speed represent faster walking with respect to the OFF condition (**A**). Positive values of DTC for gait speed represent an improvement with respect to the OFF condition (**B**). *P < 0.05. MDS‐UPDRS, Movement Disorder Society Unified Parkinson's Disease Rating Scale; DTC, dual‐task cost. [Color figure can be viewed at wileyonlinelibrary.com]

Friedman ANOVA showed a significant effect of stimulation on the change score of the Composite Motor Score (χ^2^(2) = 11.1, *P* = 0.004). Post hoc analysis showed that the change score in motor symptoms with 10 Hz stimulation was worse than with 130 Hz (*P* = 0.002) and with 130 + 10 Hz (*P* = 0.01) (Fig. 2A).

For the change score of gait velocity, Friedman ANOVA showed no significant effect of the stimulation conditions (χ^2^(2) = 2.6, *P* = 0.2) (Fig. 2B). The change score of the DTC for gait velocity did not differ among the three stimulation conditions (χ^2^(2) = 0.4, *P* = 0.8) (Fig. 2C).

### N‐Back Task and Subjective Feelings

There was no effect of the different stimulation conditions on any of the N‐back task outcomes or scores of fatigue and stress levels (Supplementary Table S1).

### Correlation Analyses

Spearman correlation analysis showed that there was no significant correlation between change score in VF in the 130 Hz or in the 130 + 10 Hz condition and age, disease duration, LEDD, MoCA, and any change score in gait and fatigue or stress levels in the same stimulation condition.

**FIG. 3:**
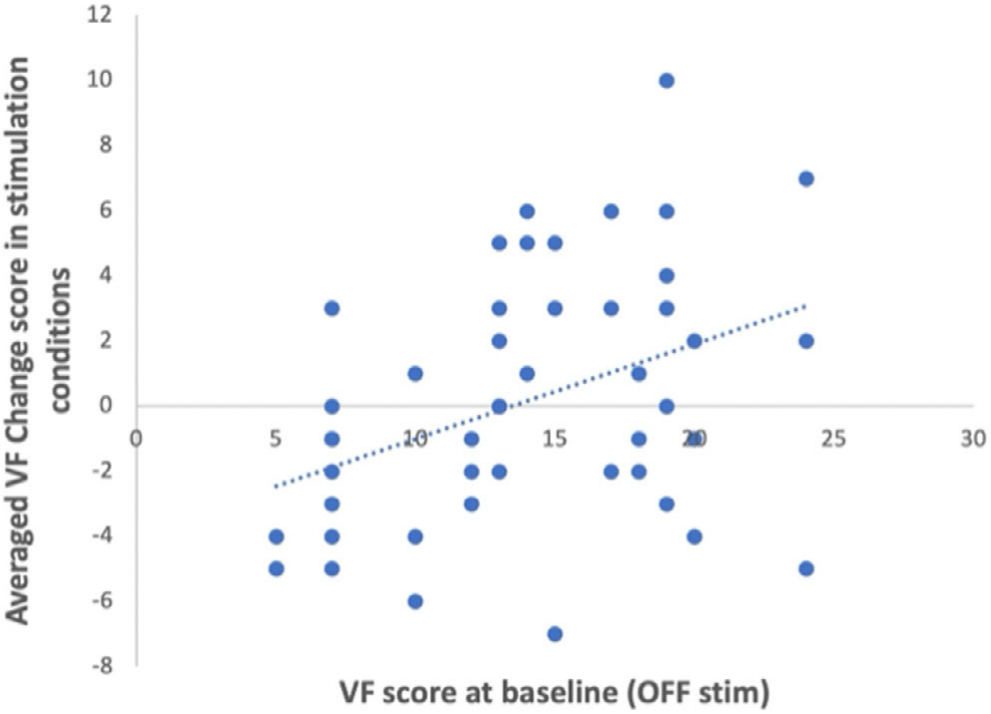
The scatter plot shows the relationship between mean stimulation‐induced changes in verbal fluency (VF) across all three conditions and VF at baseline (OFF stimulation). This indicates that lower VF at baseline is related to greater improvement with stimulation. [Color figure can be viewed at wileyonlinelibrary.com]

Moreover, there was a significant positive correlation between stimulation‐induced changes in VF performance across all frequencies and baseline VF performance (VF OFF‐stimulation) (ρ = 0.40, *P* = 0.004) (Fig. 3). This indicates that poorer VF at baseline is related to greater improvement with stimulation across conditions.

No significant correlations were observed between the VTAs associated with motor, associative, and limbic regions of left, right, and both hemispheres and VF scores across any of the stimulation conditions (all *P* > 0.05).

## Discussion

This study investigated the effects on VF of a novel DBS paradigm combining high‐frequency stimulation (130 Hz) with very low frequency stimulation (10 Hz), which were applied simultaneously over two adjacent contacts on the same lead. We compared this new paradigm to conventional 130 Hz alone, 10 Hz alone, and OFF stimulation. Aware of the known detrimental effect of very low‐frequency stimulation on motor function, we also looked at the motor effect of these stimulating paradigms. Finally, we also accounted for the potential influence of attention/working memory as well as emotional factors on VF by means of the N‐back task and visual analogue scales measuring fatigue and stress levels.

Our findings demonstrate an enhanced phonemic VF while simultaneously achieving optimal control of motor symptoms with a dual frequency DBS protocol, an approach made possible by current DBS devices able to independently control two interleaved programs running independently on each electrode. Importantly, these improvements in VF only correlated with baseline severity and were independent of any changes in attention/working memory, fatigue, and stress.

The STN is a primary target for DBS in treating the motor symptoms of PD.^2^ However, there are concerns about its effects on VF as several studies show a worsening of this domain after surgery,^3^, ^5^ supporting the notion that STN is involved in cognitive processing, especially executive functions.^34^ As a multifunctional hub, the STN is believed to mediate various non‐motor functions by using different oscillatory frequency bands.^35^ Very low frequency oscillations (θ‐α) in particular, have gained attention because of their presence in cortical and subcortical recordings during tasks involving working memory and cognitive conflict.^35^, ^36^, ^37^ Moreover, very low frequency oscillations have been suggested as physio‐markers of neuropsychiatric symptoms in PD.^35^, ^38^, ^39^

Accordingly, STN stimulation <10 Hz has been shown to improve cognitive and emotional processing in PD.^40^ Specifically, VF can improve with 10 Hz DBS, possibly by enhancing synchronization among cortical regions involved in this cognitive process.^9^, ^12^, ^13^

Interestingly, the severity of VF impairment at baseline correlated with the degree of improvement across stimulation conditions. This is in line with previous research showing that stimulation across different frequencies had a stronger positive effect on working memory in individuals with lower baseline scores,^15^ therefore, suggesting that stimulation may work compensating for cognitive deficits rather than enhancing cognitive performance.^15^

The lack of significant correlations between VTAs and VF performance in our sample suggests that the specific locations targeted by different stimulation frequencies do not significantly affect VF outcomes. Although previous studies have proposed that stimulation site influences VF performance,^12^ our findings support the idea that—as long as electrodes are within the STN–frequency‐dependent effects, rather than location‐based effects, drive changes in VF. This indicates that the effects of stimulation likely involve broader network dynamics beyond the targeted regions. Moreover, this finding suggests that VTA, although a useful tool for understanding the spatial extent of stimulation, does not fully capture the functional impact of stimulation on cognitive outcomes. For instance, the role of network connectivity and temporal dynamics of stimulation could influence cognitive performance in ways not directly reflected in the modeled VTAs. A well‐known limitation of VTA is the fact that the model does not take into account the effect of stimulation frequency, and this might have played a role in our analysis.^41^, ^42^

Despite the cognitive benefits, in previous studies, low frequency stimulation has been ineffective in treating motor symptoms—and in fact it can worsen them.^9^ Importantly, the novel dual frequency protocol presented in our study is effective in maintaining optimal motor symptom control while improving VF. Indeed, in our cohort, although 10 Hz alone did not improve motor function as compared to OFF stimulation, the combination of very low and high frequency stimulation showed the same benefits of the conventional 130 Hz stimulation used for motor symptom treatment. This has relevant clinical impact as the main limitation for using 4 to 10 Hz DBS so far has been its lack of optimal control of motor symptoms.

Although we observed this benefit on motor functions, we did not observe a significant effect of the different stimulations on gait velocity. This is in keeping with the well‐known notion that axial motor signs might take hours to change after DBS manipulations.^43^

We did not observe an effect of the different stimulation conditions on the N‐back task, a measure of working memory, which seemingly contrasts with previous findings reporting an improvement in a modified Sternberg task following 4 Hz STN‐DBS as compared to high frequency stimulation.^15^ The differing results between the N‐back and Sternberg tasks may be explained by the involvement of different cognitive demands and associated brain activation patterns in these two tasks. Indeed, the N‐back task primarily involves manipulation of information, requiring participants to update their memory with each new letter presented, whereas the Sternberg task focuses on maintenance, as participants memorize a set of letters and later identify them.^44^ Our results suggest that different STN associated brain pathways may play a different role in these two cognitive processes, with perhaps a different effect of stimulation frequency on maintenance (as seen in the Sternberg task) and manipulation of working memory (as required by the N‐back task). Moreover, it is important to note that Salehi and colleagues^15^ did not include a 10 Hz stimulation condition in their study, which introduces a key difference in experimental designs and suggests that varying stimulation frequencies can produce distinct effects on different cognitive performances such as VF and working memory.

Furthermore, our findings of enhanced VF with dual frequency stimulation compared to conventional high frequency stimulation were independent from any effect on subjective emotional state. Although previous work has reported a beneficial effect of 10 Hz stimulation on emotional processing, these studies specifically examined the effects of time‐locked acute 10 Hz stimulation of the right STN during emotional imagery tasks, which may enhance emotional processing and subjective valence.^18^, ^19^ In contrast, our study investigates the impact of bilateral STN‐DBS on subjective momentary assessments of how much patients felt stressed or fatigued, which may not capture the nuanced effects of stimulation on emotional responses in the same context.

Although this study introduces a novel dual‐frequency DBS paradigm, we recognize several limitations. First, we did not account for laterality, which may influence emotional processing, as the right hemisphere is more involved in this domain.^18^, ^19^, ^45^, ^46^, ^47^ Laterality likely plays a crucial role in VF, which is mainly left hemisphere‐dependent. Recent studies show that VF declined after left STN DBS, but improved after right STN DBS,^48^ with declines in VF linked to the position of the left electrode along the dorsal‐ventral axis.^49^ Second, the sample size was small, although it is comparable to previous studies and still enabled us to detect a significant effect.

Third, the relatively short observation period and wash‐out time, which may have contributed to a carry‐over effect and limited the ability to detect changes in motor function, particularly in the 10 Hz condition. However, this waiting time was longer than that used in most previous studies.^12^, ^13^, ^15^ Additionally, the use of the MDS‐UPDRS may not have been sensitive enough to capture finer motor changes, and a more detailed kinematic assessment would be needed in future studies to address this. Finally, the lack of brain connectivity data does not clarify the brain networks involved in the different frequency effects, possibly related to aforementioned limitations of VTA modeling when it comes to frequency adjustments.

In conclusion, we show that simultaneous very low‐ and high‐frequency STN‐DBS improves VF while maintaining optimal motor control in PD patients. Future research should prioritize randomized controlled trials examining the long‐term effects of dual‐frequency stimulation versus high‐frequency DBS, using real‐life outcomes. Finally, from a mechanistic standpoint, future studies might benefit from a more targeted exploration of cortical and subcortical networks in relation to stimulation parameters and to the excitatory or inhibitory effects of different DBS frequencies on cognitive networks.

## Author Roles

(1) Research project: A. Conception, B. Organization, C. Execution; (2) Statistical Analysis: A. Design, B. Execution, C. Review and Critique; (3) Manuscript Preparation: A. Writing of the First Draft, B. Review and Critique.

L.R.: 1A, 1B, 2A, 2B, 3A

F.P.C.: 1C

E. Pegolo: 1C, 2B

A.A.C.: 1C, 3B

B.I.: 1C, 2B

I.H.: 1B

Z.S.: 3B

M.H.: 3B

E.Pereira: 3B

F.M.: 1B, 3B

A.F.: 1A, 1B, 3B

## Financial Disclosures

This research was supported by Clinical Academic Research Partnerships Medical Research Council/National Institute for Health and Care Research (CARP MRC/NIHR) grant under award number MR/T023864/1 (L.R.) and the University of Toronto/University Health Network Chair in Neuromodulation (A.F.). L.R. served in the advisory board of Boston Scientific and Britannia; and she received speaking Honoraria from Bial and the International Parkinson's Disease and Movement Disorders Society. F.P.C., E. Pereira, A.A.C., B.I., and I.H. do not report conflict of interest. M.H. is a member of the Medicines and Healthcare products Regulatory Agency (MHRA) interim devices working group. E. Pegolo receives speaking honoraria from Boston Scientific and Medtronic; and royalties from Elsevier and Oxford University Press. F.M. received consultancy fees from Boston Scientific and Medtronic; has been/is a member of advisory boards of AbbVie, Boston Scientific, Merz, Medtronic, and Roche; and received speaking Honoraria from AbbVie, Boston Scientific, Merz, Medtronic, and the International Parkinson's Disease and Movement Disorders Society. She has received research support by NIHR and Innovate UK; and receives royalties from Springer. A.F. has stock ownership in Inbrain Pharma; and has received payments as consultant and/or speaker from AbbVie, Abbott, Boston Scientific, Ceregate, Dompé Farmaceutici, Inbrain Neuroelectronics, Ipsen, Medtronic, Iota, Syneos Health, Merz, Sunovion, Paladin Labs, UCB, and Sunovion. He has received research support from AbbVie, Boston Scientific, Medtronic, Praxis, and ES; and receives royalties from Springer.

## Supporting information


**Data S1.** Supporting Information.

**Table S3.** Table information.

## Data Availability

The data that support the findings of this study are available on request from the corresponding author. The data are not publicly available due to privacy or ethical restrictions.
