# Supplementary material for: Low/High Multi‐Frequency Stimulation of the Subthalamic Nucleus Improves Verbal Fluency Maintaining Motor Control in Parkinson's Disease
Source: Mov Disord. 2025 Jun 11;40(9):1892–900. doi: 10.1002/mds.30254 (PMC12485583; doi:10.1002/mds.30254)
Supplement: Supplementary file 1 — Data S1. Supporting Information. [file MDS-40-1892-s001.docx]

**Supplementary data**

**Supplementary** **Figures**

*Supplementary Figure 1: Study protocol*

**
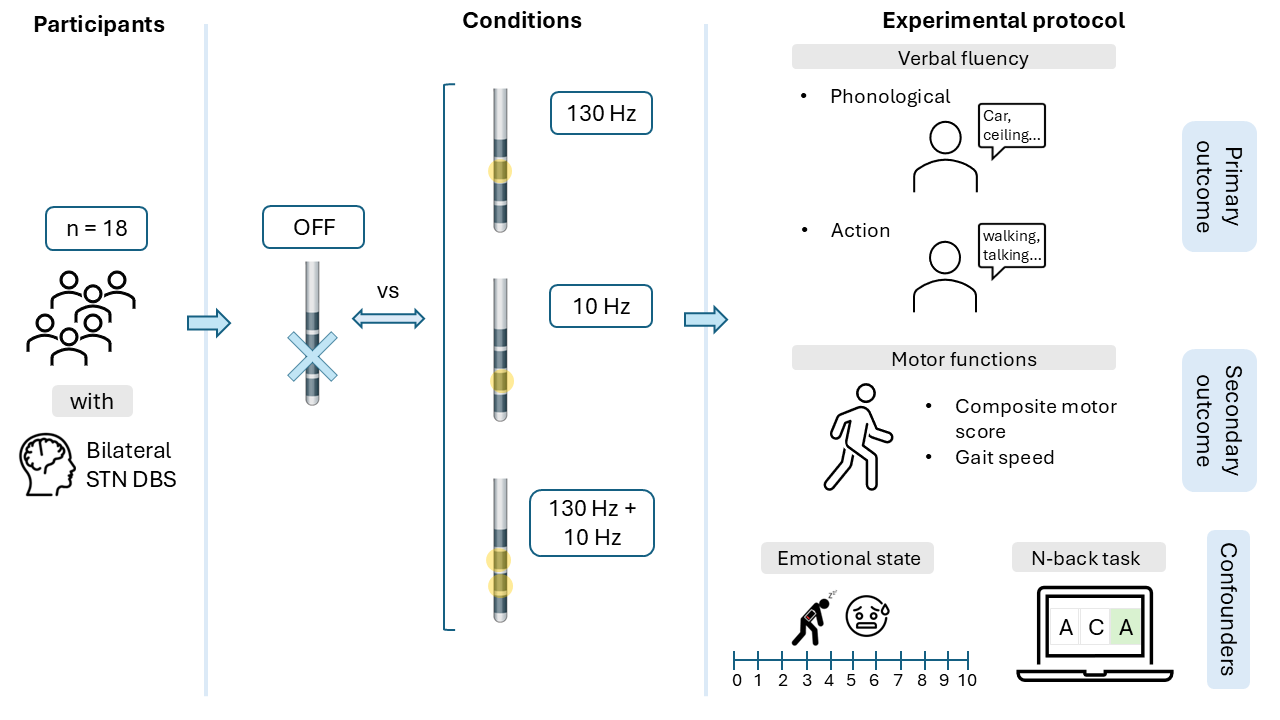
**

***Supplementary Figure 2:*** Coronal (left) and axial (right) views of electrode lead locations in MNI space for the London (A) and Toronto (B) cohorts. Showing the Distal Minimal Atlas (Ewert 2017, orange = STN sensorimotor, blue = STN associative and yellow = STN limbic).

***Supplementary Figure 3:*** *No difference in the number of words produced across different letters in the phonemic verbal fluency tasks.*


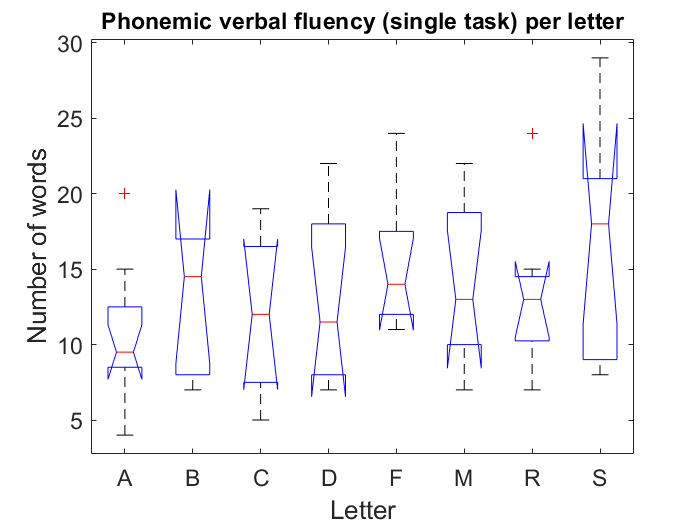

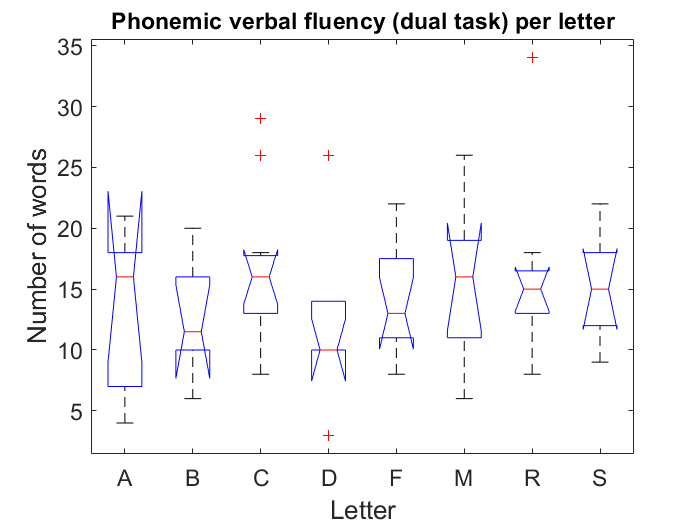


**Supplementary Tables**

*Supplementary Table 1: Demographic and clinical data of the study population*

|  | Sample (N =18) |
| --- | --- |
| Age (years) | 62.2 ± 7.4 |
| Gender | 4 F / 14M |
| Handness (R/L) | 16/2 |
| English mother tongue (Yes/No) | 15/3 |
| Disease duration (years) | 15.7 ± 5.4 |
| Tremor dominant/akinetic-rigid | 6/12 |
| LEDD tot (mg/day) | 745.2 ± 399.5 |
| DA-LEDD (mg/day) | 52.5 ± 72.9 |
| Time since DBS surgery (months) | 47.2 ± 17.7 |
| MoCA | 27 ± 1.8 |

Abbreviations: DA: dopamine-agonists; DBS: Deep Brain Stimulation; LEDD: levodopa equivalent daily dose, MoCA: Montreal Cognitive Assessment, R: right, L: left. Values are reported as mean + standard deviation.

**Supplementary Table 2: Absolute Values for the cognitive, emotional and motor scores in the 4 experimental conditions**

|  | **130 Hz** | **OFF** | **10 Hz** | **130+10 Hz** |
| --- | --- | --- | --- | --- |
| **Phonemic VF** | 11.9 ± 3.9 | 13.7 ± 5.5 | 13.6 ± 5.6 | 14.2 ± 6.0 |
| **Action VF** | 12.2 ± 5.8 | 11.5 ± 7.3 | 13 ± 7.0 | 13.0 ± 7.5 |
| **N-back task Correct** | 81.6 ± 9.1 | 80.4 ± 11.3 | 81.6 ± 10.9 | 83.0 ± 11.6 |
| **N-back task Match** | 72.6 ± 17.2 | 67.8 ± 24.5 | 73.9 ± 21.6 | 72.3 ± 21.7 |
| **N-back task False Alarm** | 15.4 ± 11.2 | 13.1 ± 9.5 | 14.8 ± 10.6 | 12.1 ± 11.9 |
| **Tiredness** | 4.6 ± 2.6 | 4.6 ± 2.3 | 5.5 ± 2.0 | 5.1 ± 2.0 |
| **Stress** | 6.3 ± 2.0 | 4.1 ± 2.4 | 5.5 ± 2.1 | 7.1 ± 1.4 |
| **Composite Motor Score** | 3.9 ± 2.3 | 8.2 ± 3.5 | 5.6 ± 2.6 | 4.1 ± 2.5 |

Mean ± standard deviation of the absolute values for VF (number of words), N-back task (% of total), emotional state (visual analog scale from 0 to 10) and Composite Motor Score. Composite Motor score is the sum of MDS-UPDRS items 3.4 (finger tapping), 3.10 (gait) and 3.17 (Resting Tremor). Abbreviations: MDS-UPDRS: Movement Disorder Society Unified Parkinson’s Disease Rating Scale. VF: Verbal Fluency.

*Supplementary Table 4: Change scores in N-back task performance, action fluency and emotional states in the different stimulation conditions.*

|  | 130 Hz | 10 Hz | 130+10 Hz | Chi-squared | p value |
| --- | --- | --- | --- | --- | --- |
| Subjective Feelings | | | | | |
| Tiredness | 0.4 ± 1.6 | -0.8 ± 2.2 | -0.4 ± 2.6 | 2.8 | 0.2 |
| Stress | -2.3 ± 2.0 | -1.4 ± 1.7 | -3.0 ± 2.6 | 5.4 | 0.06 |
| Cognitive tasks | | | | | |
| Action Fluency | -0.4 ± 5.4 | -1.6 ± 4.4 | -1.6 ± 5.9 | 0.7 | 0.7 |
| Correct | -1.2 ± 7.8 | -1.2 ± 9.4 | -2.6 ± 10.5 | 1.4 | 0.5 |
| Match | -4.8 ± 20.0 | -6.0 ± 17.2 | -4.5 ± 17.0 | 0.4 | 0.8 |
| False alarm | -2.2 ± 7.0 | -1.7 ± 6.4 | 1.0 ± 9.3 | 3.4 | 0.2 |

Mean ± standard deviation of the change scores in emotional states ratings, action verbal fluency and N-back task (correct, match, and false alarm) data in the conditions 130 Hz, 10 Hz, and 130+10 Hz with respect to the OFF stimulation one. Results of Friedman ANOVA test for each output are reported as chi-square and p-value.
