# Supplementary material for: Low/High Multi‐Frequency Stimulation of the Subthalamic Nucleus Improves Verbal Fluency Maintaining Motor Control in Parkinson's Disease
Source: Mov Disord. 2025 Jun 11;40(9):1892–900. doi: 10.1002/mds.30254 (PMC12485583; doi:10.1002/mds.30254)
Supplement: Supplementary file 2 — Table S3. Table information. [file MDS-40-1892-s002.pdf]

		Amplitude		Pulse width		Frequency		Configuration	
ID subject	Model	Left	Right	Left	Right	Left	Right	Left	Right
Subject_01	Vercise™ directional lead, Boston Scientific	4.1 mA	5.2 mA	40 us	50us	130 Hz	130 Hz	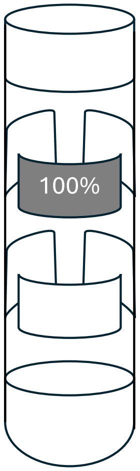	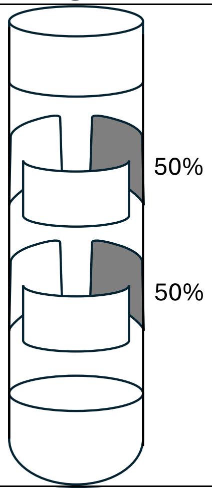
Subject_02	Vercise™ directional lead, Boston Scientific	2.2 mA	5.4 mA	50 us	60 us	130 Hz	130 Hz	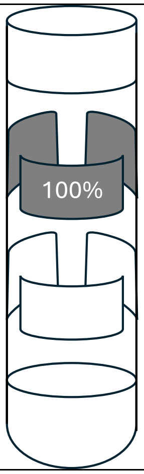	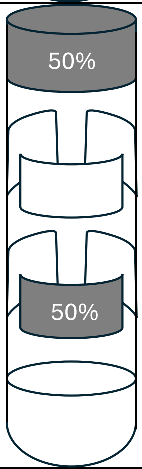
Subject_03	Vercise™ directional lead, Boston Scientific	3.7 mA	1.5 mA	60 us	40 us	130 Hz	130Hz	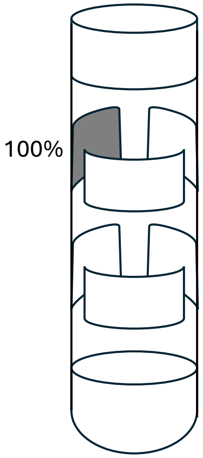	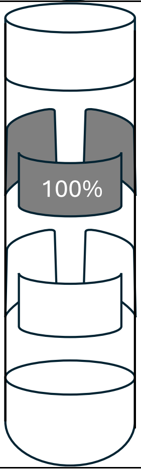

Subject_04	Cartesia™ X lead, Boston Scientific	2.9 mA	3.5 mA	60 us	40 us	130 Hz	130 Hz	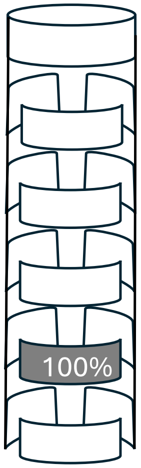	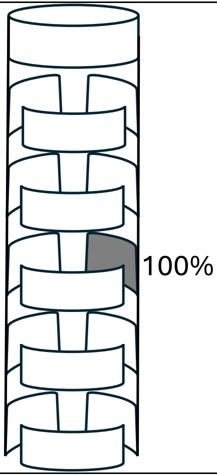
Subject_05	Vercise™ standard lead, Boston Scientific	4.3 mA	4.2 mA	60 us	60 us	130 Hz	130 Hz	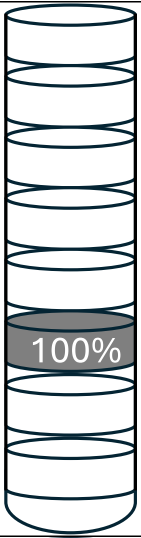	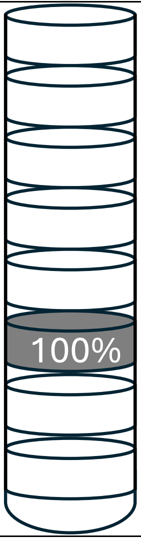
Subject_06	Vercise™ directional lead, Boston Scientific	3.4 mA	2.2 mA	60 us	60 us	179 Hz	179 Hz	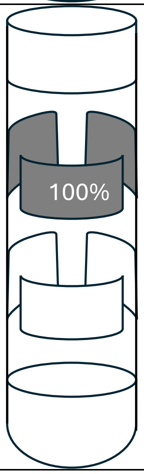	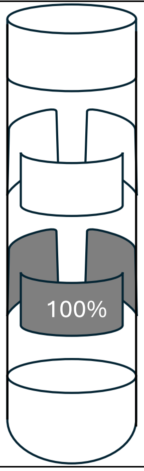

Subject_07	Vercise™ directional lead, Boston Scientific	2.7 mA	3.0 mA	50 us	30 us	130 Hz	130 Hz	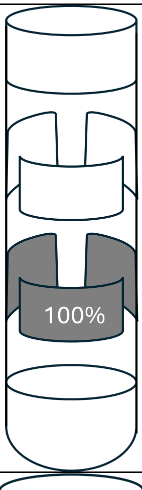	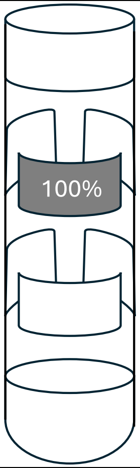
Subject_08	Vercise™ directional lead, Boston Scientific	3.0 mA	2.6 mA	60 us	60 us	130 Hz	130 Hz	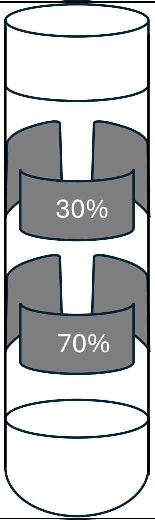	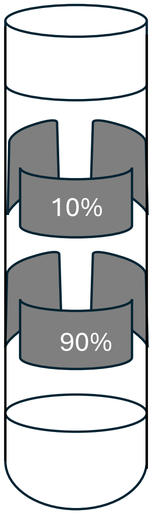
Subject_09	Vercise™ directional lead, Boston Scientific	2.7 mA	3.0 mA	60 us	40 us	130 Hz	130 Hz	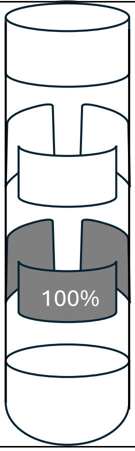	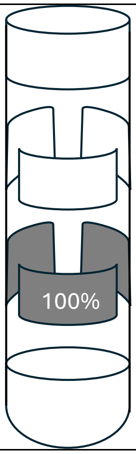

Subject_10	Vercise™ standard lead, Boston Scientific	3.7 mA	3.5 mA	60 us	60 us	130 Hz	130 Hz	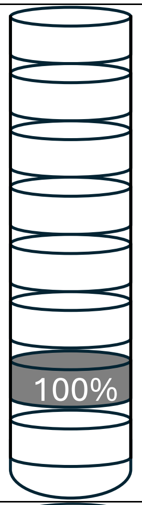	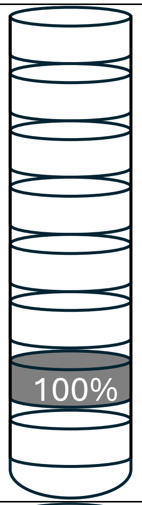
Subject_11	Vercise™ directional lead, Boston Scientific	1.8 mA	2.6 mA	50 us	60 us	130 Hz	130 Hz	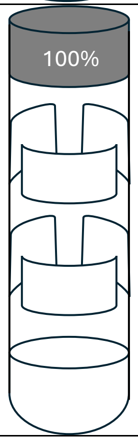	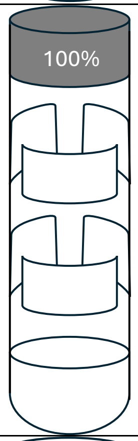
Subject_12	Vercise™ directional lead, Boston Scientific	2.1 mA	3.7 mA	50 us	50 us	130 Hz	130 Hz	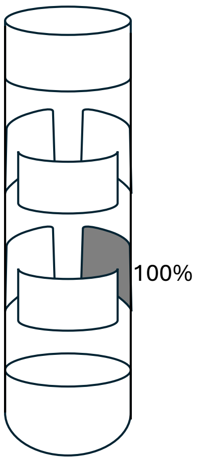	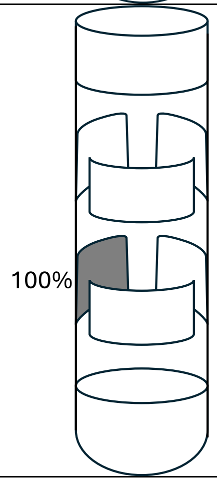

Subject_13	Vercise™ directional lead, Boston Scientific	2.5 mA	2.0 mA	60 us	50 us	130 Hz	130 Hz	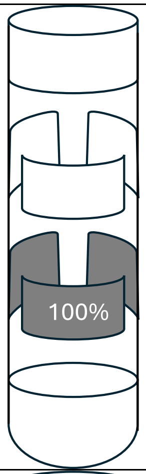	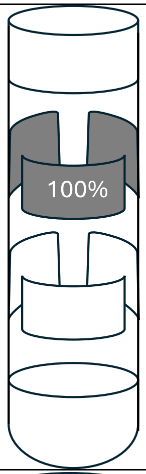
Subject_14	Vercise™ directional lead, Boston Scientific	2.9 mA	2.9 mA	60 us	60 us	130 Hz	130 Hz	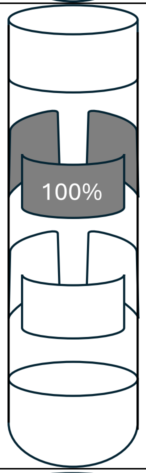	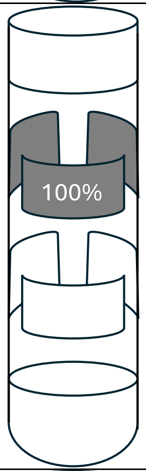
Subject_15	Vercise™ directional lead, Boston Scientific	4.7 mA	4.0 mA	40 us	60 us	130 Hz	130 Hz	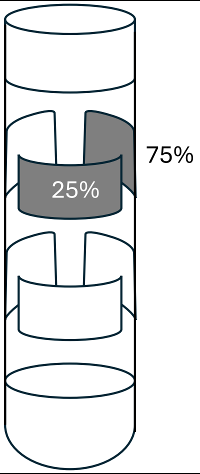	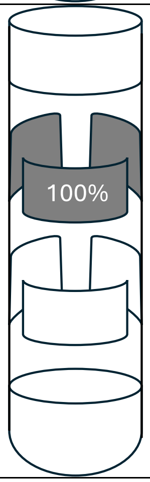

Subject_16	Vercise™ directional lead, Boston Scientific	5.0 mA	6.5 mA	60 us	40 us	130 Hz	130 Hz		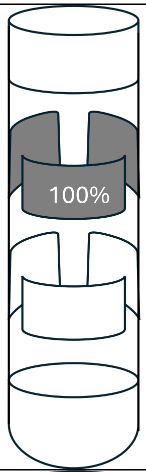	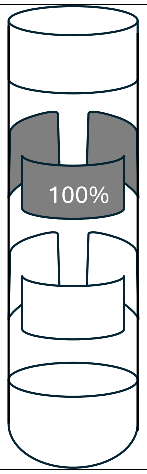
Subject_17	Vercise™ directional lead, Boston Scientific	2.6 mA	3.8 mA	60 us	60 us	130 Hz	130 Hz		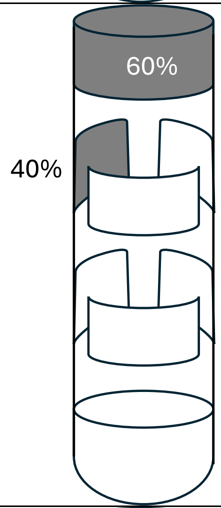	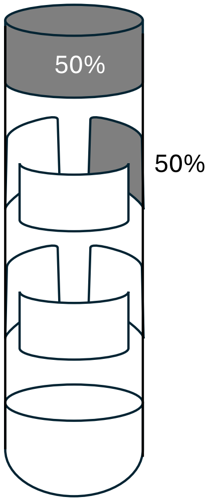
Subject_18	Vercise™ directional lead, Boston Scientific	6.5 mA	6.3 mA	40 us	40 us	130 Hz	130 Hz		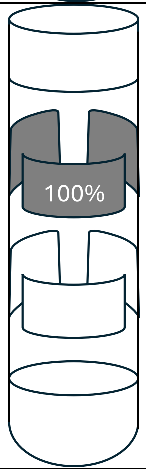	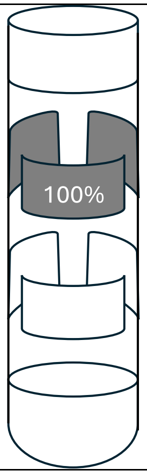
